# Preclinical evaluation of pentagamavunone-1 as monotherapy and combination therapy for pancreatic cancer in multiple xenograft models

**DOI:** 10.1038/s41598-022-26863-y

**Published:** 2022-12-27

**Authors:** Naoki Kamitani, Ikuko Nakamae, Noriko Yoneda-Kato, Jun-ya Kato, Masayuki Sho

**Affiliations:** 1 Department of Surgery, Nara Medical University, 840 Shijo-Cho, Kashihara, Nara 634-8522 Japan; 2 Laboratory of Tumor Cell Biology, Division of Biological Science, Graduate School of Science and Technology, Nara Institute of Science and Technology, 8916-5, Takayama, Ikoma, Nara 630-0101 Japan

**Keywords:** Developmental biology, Cancer, Drug development, Medical research, Experimental models of disease, Preclinical research

## Abstract

We previously reported that pentagamavunone-1 (PGV-1) effectively inhibited cell proliferation in many types of human tumors, including pancreatic cancer, by inducing M phase (prometaphase) arrest, senescence, and apoptosis with few side effects. However, a detailed evaluation of the effects of PGV-1 on pancreatic cancer cells in an in vivo setting has not yet been conducted. The present study investigated the potential efficacy of PGV-1 as both monotherapy and combination therapy for pancreatic cancer using multiple xenograft mouse assays. A cell-line derived xenograft model (CDX-M) with pancreatic cancer cell line and a patient-derived xenograft mouse model (PDX-M) using resected pancreatic cancer samples without neoadjuvant chemotherapy were established in both heterotopic and orthotopic manners. PGV-1 effectively suppressed tumor formation at the heterotopic and orthotopic sites in CDX-M than in untreated mice. Combination therapy with PGV-1 and gemcitabine more effectively suppressed tumor formation than monotherapy with PGV-1 or gemcitabine when administered after tumor formation. Monotherapy with PGV-1 or gemcitabine less effectively suppressed tumor formation in PDX-M than in CDX-M, whereas combination therapy with PGV-1 and gemcitabine more effectively suppressed tumor formation. PGV-1 as monotherapy and combination therapy with gemcitabine effectively inhibited tumor formation and has potential as an anticancer candidate for pancreatic cancer.

## Introduction

Pancreatic cancer is one of the most aggressive types of human cancer. The 5-year overall survival rate is still approximately 9%, which is the lowest among all human cancers^1–3^. Since most cases are diagnosed at an advanced stage, chemotherapy and radiation therapy are the only treatment options for 85% of patients^4,5^. Among many chemotherapeutic agents, gemcitabine is a key drug for pancreatic cancer; however, it is not sufficiently effective and its response rate was previously estimated to be approximately 10% when used as monotherapy^5,6^. The causes of ineffectiveness include resistance to gemcitabine. In clinical cases, patients with advanced pancreatic cancer receive combination chemotherapy with gemcitabine and nab-paclitaxel as a first-line regimen^7^. This combination therapy has been shown to increase overall survival to 8.5 months from 5.7 months with gemcitabine monotherapy^6^. One of the limitations of chemotherapeutic drugs is side effects; gemcitabine exerts anti-tumor effects by inhibiting the replication of DNA in tumor cells at the S phase of the cell cycle^8^, which also damages normal cells, resulting in myelosuppression. Similarly, taxanes, the main component of nab-paclitaxel, inhibit the effects of microtubulin in the M phase, which prevents axonal transport in neurons, resulting in neurotoxicity^9^. The findings of a clinical trial demonstrated that the rates of severe neutropenia and peripheral neuropathy were 38 and 17%, respectively^6^. Severe side effects interrupt cancer treatment even when sufficient therapeutic effects are achieved, and, thus, there is a strong need for innovative agents with fewer side effects.

We previously reported the novel aspects of a new chemotherapeutic compound, pentagamavunone-1 (PGV-1). Although PGV-1 was originally developed as one of the related products of curcumin^10^, it exhibited more distinctive activity than curcumin; it exerted strong cytotoxic effects against various cancer cell lines by interrupting the progression of the cell cycle at the M phase (prometaphase)^11–13^, and few side effects were observed due to markedly weaker effects on normal cells. PGV-1 also exhibited anti-tumor efficacy as combination therapy with another compound and as monotherapy in vitro^14,15^. However, many questions remain unanswered due to the lack of in vivo data^13^. Therefore, we decided to investigate the in vivo efficacy of PGV-1 using pancreatic cancer cells, thereby providing insights into the potential of PGV-1 as a novel chemotherapeutic drug.

The use of preclinical models is a crucial step in every aspect of translational cancer research. Many candidates for anti-cancer drugs are investigated in cell culture experiments using cell lines and a cell line-derived xenograft mouse model (CDX-M) as preclinical models in translational research on oncology. However, the high ratio of failure by novel anti-cancer candidates in clinical trials partly results from the difficulties associated with experiments using cell lines to predict clinical efficacy^16^. One of these difficulties is the microenvironment surrounding cancer tissue in addition to the heterogeneity of cancer cells included in cancer tissue. The microenvironment of pancreatic cancer cells has two prominent features: the lack of vessels and richness in a dense stroma. The lack of vessels in pancreatic cancer tissue is associated with poor drug delivery, which limits the ability of any systemically administered drug to penetrate tumor cells and reduces perfusion^17^. A dense stroma, consisting of inflammatory and immune cells, endothelial cells, and the extracellular matrix, forms a physical barrier to chemotherapeutic agents penetrating tumor cells^18^. Furthermore, established cell lines are immortalized cells that have different phenotypes and genotypes from tumor cells in patients^19,20^. Therefore, data obtained from experiments using cell lines may not necessarily reflect efficacy in actual cases. A patient-derived xenograft mouse model (PDX-M) has been employed to overcome these difficulties. The heterogeneity, histological features, and microenvironments of tumor cells are more accurately preserved in PDX-M than in CDX^21,22^. Therefore, PDX-M is a better platform to verify the potential efficacy of anti-cancer drugs for pancreatic cancer.

In the present study, we performed a preclinical evaluation of PGV-1 as monotherapy and combination therapy with gemcitabine in multiple xenograft mouse models using pancreatic cancer cells. Implantation was performed at orthotopic and heterotopic sites, and a patient-derived orthotopic mouse model (PDOX) was also established. The advantages of PDOX are that tumor cells represent the appropriate sites of organs for human cancers, and the efficacy of candidates may be examined in more detail^23,24^.

## Results

### Efficacy of PGV-1 and gemcitabine in pancreatic cancer cells in vitro

**Figure 1:**
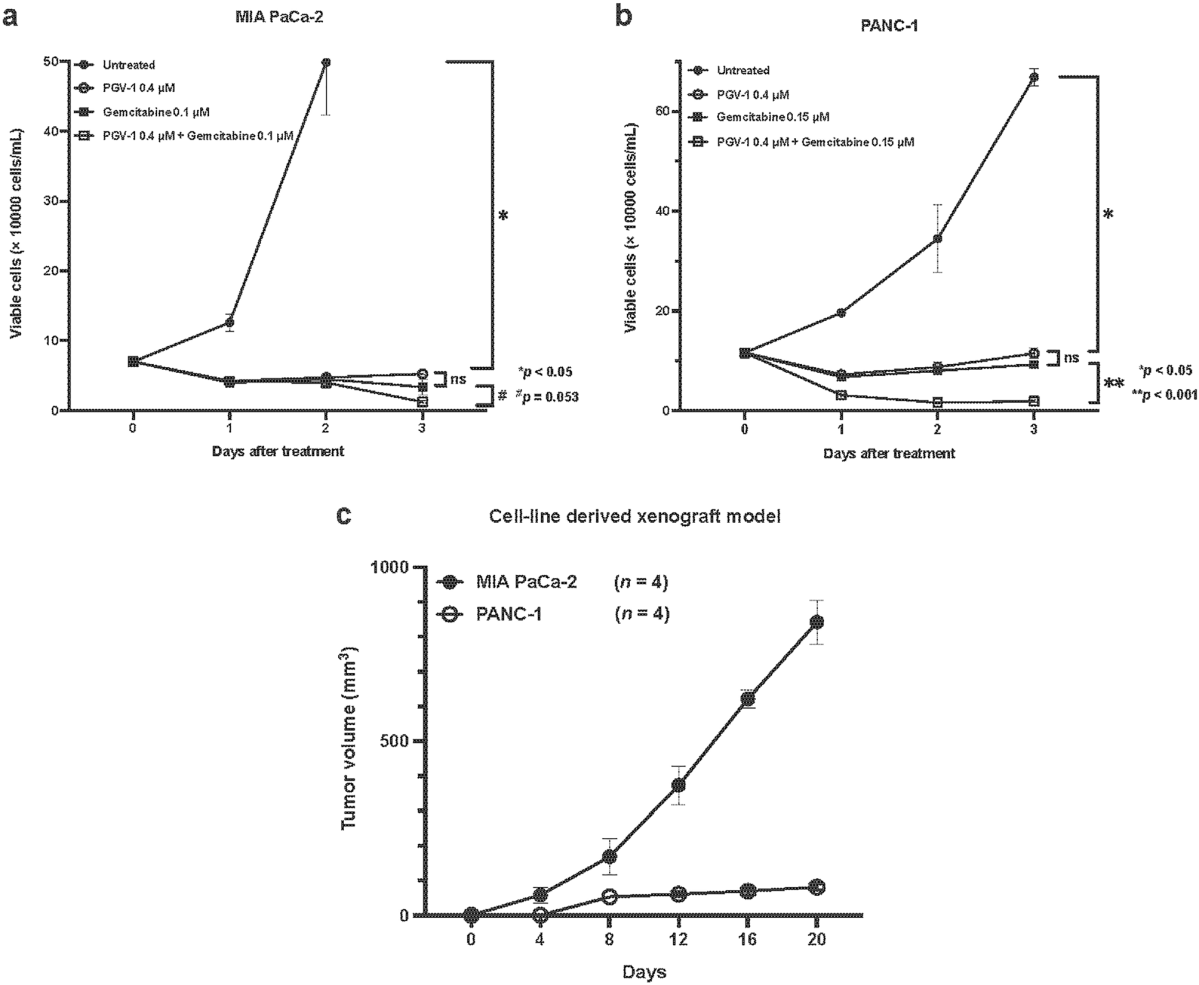
PGV-1 suppresses pancreatic cancer cell proliferation in vitro. MIA PaCa-2 (**a**) and PANC-1 (**b**) cells were cultured with PGV-1 (0.4 μM), gemcitabine (0.10 and 0.15 μM), and a combination of PGV-1 (0.4 μM) and gemcitabine (0.10 and 0.15 μM) for 3 days. Viable cells were counted by the trypan blue exclusion method at the indicated time. P values were calculated with Student’s t test. The results are shown as the average of 4 independent experiments (means ± SEM). (**c**) MIA PaCa-2 and PANC-1cells (1 × 10^7^ cells) were subcutaneously injected into nude mice, and tumor volumes were measured every 2 days. The results are shown as the average of 4 independent experiments (one mouse per one independent experiment. means ± SEM).

We investigated the anti-tumor effects of PGV-1 using MIA PaCa-2 and PANC-1 cells in comparison with gemcitabine and their combination in an in vitro cell culture system. We cultured MIA PaCa-2 and PANC-1 cells in the absence and presence of PGV-1 (0.4 μM) with and without gemcitabine (0.1–0.15 μM) in vitro and counted viable cells for three days (Fig. 1a,b). In comparisons with untreated cells, PGV-1 and gemcitabine each markedly suppressed the growth of MIA PaCa-2 and PANC-1 cells to a similar extent. The combination of PGV-1 and gemcitabine more effectively suppressed cell growth than PGV-1 or gemcitabine alone. These results suggested that PGV-1 was effective in CDX-M using pancreatic cancer cells. Our preliminary experiment revealed that tumor formation by PANC-1 cells was very slow in CDX-M (Fig. 1c); therefore, we used MIA PaCa-2 cells to investigate the efficacy of PGV-1 in CDX-M.

### Antitumor efficacy of PGV-1 in a CDX-M assay using pancreatic cancer cells

**Figure 2:**
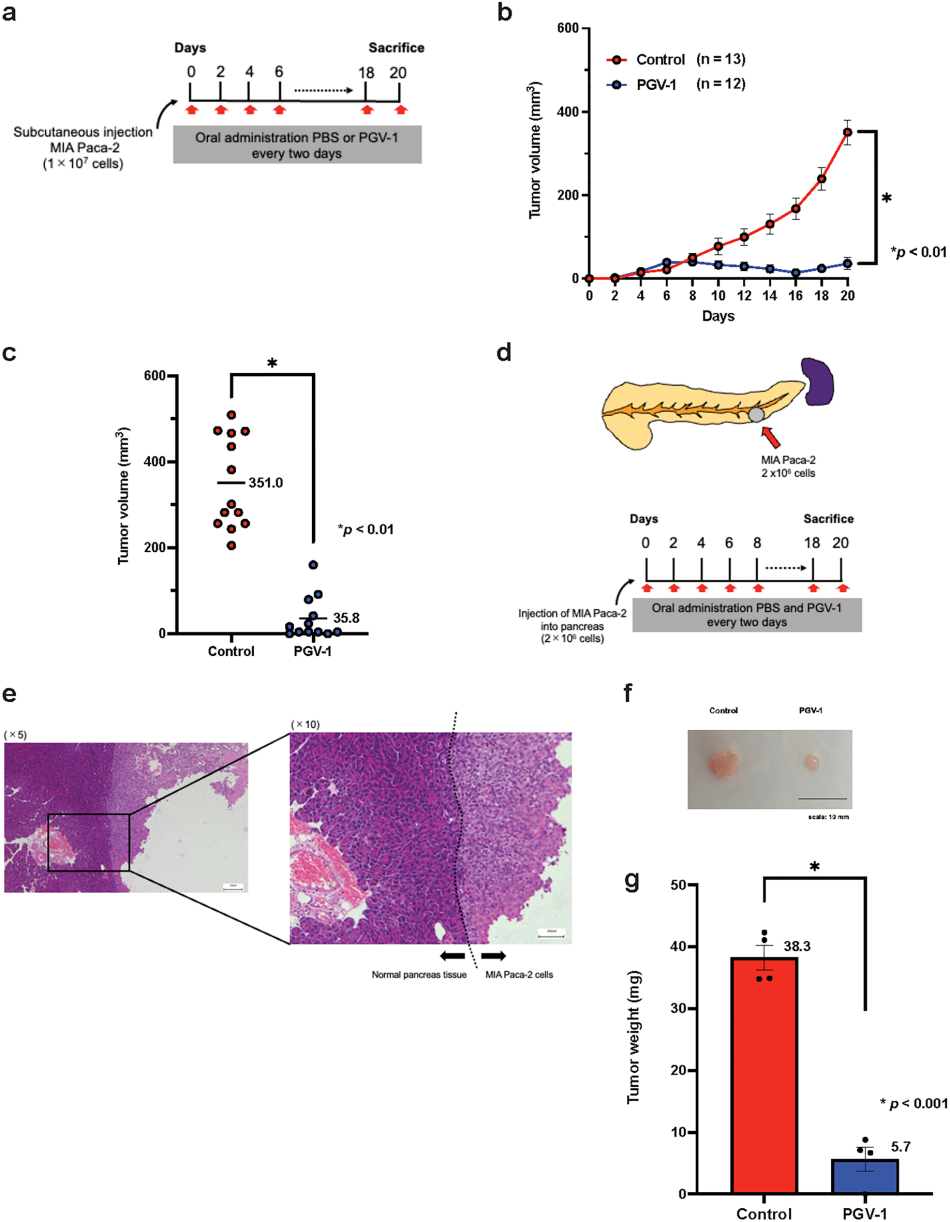
PGV-1 suppresses tumor formation at both heterotopic and orthotopic sites in vivo. (**a**) Experimental design to test the anti-tumor effects of PGV-1 in CDX-M at the heterotopic site. (**b**,**c**) MIA PaCa-2 cells (1 × 10^7^ cells) were subcutaneously injected into nude mice, and PBS (control) and PGV-1 (25 mg/kg BW) in corn oil were orally administered every 2 days. Tumor volumes were measured every 2 days (**b**). On the 20th day, mice were sacrificed and tumor volumes were measured (c). (**d**) Experimental design to test the anti-tumor effects of PGV-1 in CDOX. (**e**–**g**) MIA PaCa-2 cells (2 × 10^6^ cells) were injected into the pancreas of nude mice, and PBS (control) and PGV-1 (25 mg/kg BW) in corn oil were orally administered every 2 days. After 20 days, mice were sacrificed, tumors were resected, stained with HE, and observed under a microscope (**e**). Photos of actual tumors (f) and the weights of tumors (**g**) are shown. P values were calculated with Student’s t test. Data are the average of 4 independent experiments (**b**,**c**,**g**) shown as mean ± SEM (one to four mice were used for each independent experiment).

We previously demonstrated that PGV-1 suppressed tumor growth in a xenograft mouse model using leukemic K562 cells. Since an in vivo assessment of PGV-1 has not yet been performed using pancreatic cancer cell lines, MIA PaCa-2 pancreatic cancer cells were employed to examine PGV-1 in the same subcutaneous xenograft mouse model and oral administration system as those used for leukemic cells. MIA PaCa-2 cells (1 × 10^7^ cells) were subcutaneously injected and PGV-1 or PBS was then orally administered to these mice (Fig. 2a). After 20 days, MIA PaCa-2 cells formed tumors with an average size of 351.0 mm^3^, whereas tumors were markedly smaller in PGV-1-treated mice; tumorigenicity decreased to 3.4% (10.3 mm^3^ *vs.* 301.4 mm^3^; *p* < 0.01) and TGI for PGV-1 was 96.6% (Fig. 2b,c). Therefore, PGV-1 inhibited tumor formation by pancreatic cancer cells in vivo when orally administered, as previously reported for K562 cells.

To further investigate the anti-tumor efficacy of PGV-1, a cell line-derived orthotopic model (CDOX) was established. An orthotopic transplantation model allows tumor cells to behave in a similar manner to pancreatic cancer in patients, and more accurately reflects actual treatment for pancreatic cancer. MIA PaCa-2 cells (2 × 10^6^ cells) were injected into the pancreas under the pancreatic coating capsule. Similar to the experiment on subcutaneous tumors, PGV-1 or PBS was then orally administered to these mice (Fig. 2d). After 20 days, mice were sacrificed and tumors were resected. HE-stained tumor samples showed that the boundary between normal pancreatic tissue and MIA PaCa-2 cells was clear, and MIA PaCa-2 cells were located between normal pancreatic acinar cells (Fig. 2e), indicating that MIA PaCa-2 cells were injected in a technically precise manner. The mean tumor weight was 38.3 ± 4.0 mg. When PGV-1 was administered, tumorigenicity decreased to 14.9% (5.7 mg *vs.* 38.3 mg; *p* < 0.001) and TGI for PGV-1 was 85.1% (Fig. 2f,g). These results indicated that PGV-1 exerted anti-tumor effects in vivo at the orthotopic and heterotopic sites following its oral administration.

### PGV-1 blocked the progression of the M phase and induced apoptosis in tumor cells in vivo

**Figure 3:**
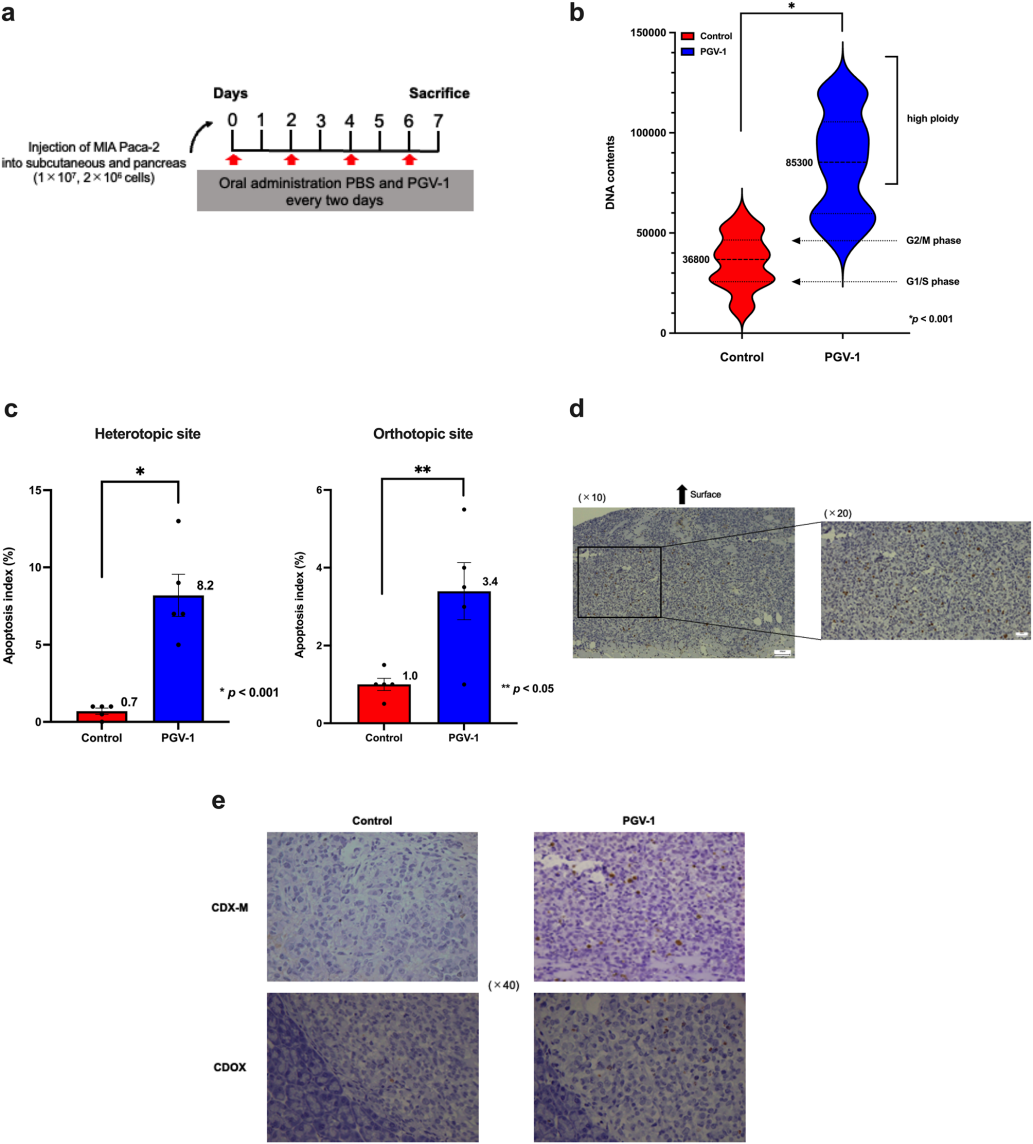
PGV-1 blocks progression of the M phase and induces apoptosis in vivo. (a) Experimental design to test the effects of PGV-1 in vivo. (**b**–**e**) MIA PaCa-2 cells were injected subcutaneously (1 × 10^7^ cells) and into the pancreas (2 × 10^6^ cells) of nude mice, and PBS (control) and PGV-1 (25 mg/kg BW) were orally administered every 2 days. On day 7, mice were sacrificed and tumors were resected. (**b**) Tumors were cut into small pieces in medium, and tumor cells were filtered, fixed onto glass slides by the cytospin technique, and stained with Hoechst 33342. Chromosomal DNA was viewed under confocal microscopy and DNA contents were measured. (**c**) Apoptosis indices were calculated at both the heterotopic and orthotopic sites and compared between the control and PGV-1 groups (Left panel; heterotopic site, Right panel; orthotopic site). (**d**) Microscopic image of the tumor after TUNEL staining in the PGV-1 group. (**e**) Comparison of microscopic images of tumors after TUNEL staining in CDX-M and CDOX between the control and PGV-1 groups (Left upper panel; CDX-M in the control group, Right upper panel; CDX-M in the PGV-1 group, Left lower panel; CDOX in the control group, Right lower panel; CDOX in the PGV-1 group). P values were calculated with Student’s t test. Data are the average of 2 independent experiments (**b**,**c**) shown as mean ± SEM.

We previously demonstrated that K562 leukemic cells treated with PGV-1 rapidly increased the population in the G2/M phase within 24 h followed by an increase in ploidy higher than 4n and cell death. However, these findings were obtained from an in vitro experiment, and in vivo experimental data is very limited. Therefore, we examined the parameters of the cell cycle and apoptosis in MIA PaCa-2 cells transplanted into mice and treated with PGV-1; experiments, including measurements of DNA contents and TUNEL staining, were performed. Since Fig. 2b showed that the tumor volume of CDX-M in the PGV-1 group started to decrease six days after the administration of PGV-1, we examined tumors on the 7th day after the administration of PGV-1 (Fig. 3a). DNA contents were significantly higher (4n or higher) in cells treated with PGV-1 than in the control group (Fig. 3b), indicating that PGV-1 blocked progression through the M phase in vivo, resulting in an increase in ploidy, which confirmed that the effects of PGV-1 in vivo were the same as those in vitro.

Tumor sections at heterotopic and orthotopic sites were subjected to TUNEL staining in order to assess apoptosis induced by PGV-1. As expected, more TUNEL-positive cells were observed in tumors from mice treated with PGV-1. The apoptosis index at the heterotopic site was higher in the PGV-1 group than in the control group (8.2 ± 3.0% *vs.* 0.7 ± 0.4%; *p* < 0.001) (Fig. 3c). The apoptosis index at the orthotopic site was also higher in the PGV-1 group than in the control group (3.4 ± 1.6% *vs.* 1.0 ± 0.4%; *p* < 0.05) (Fig. 3c). The histological findings of tumors at the heterotopic site in the PGV-1-treated group showed TUNEL-positive cells at both the surface and in the central part of tumor sections (Fig. 3d). Figure 3e shows representative histological findings of both the heterotopic and orthotopic sites. Collectively, these results demonstrated that the effects of PGV-1 in vivo were similar to those in vitro; PGV-1 inhibited progression through the M phase, resulting in an increase in the population of cells with ploidy higher than 4n, which triggered the induction of apoptosis possibly through a mitotic catastrophe.

### Effects of PGV-1 on pre-formed tumors in the CDX-M assay

**Figure 4:**
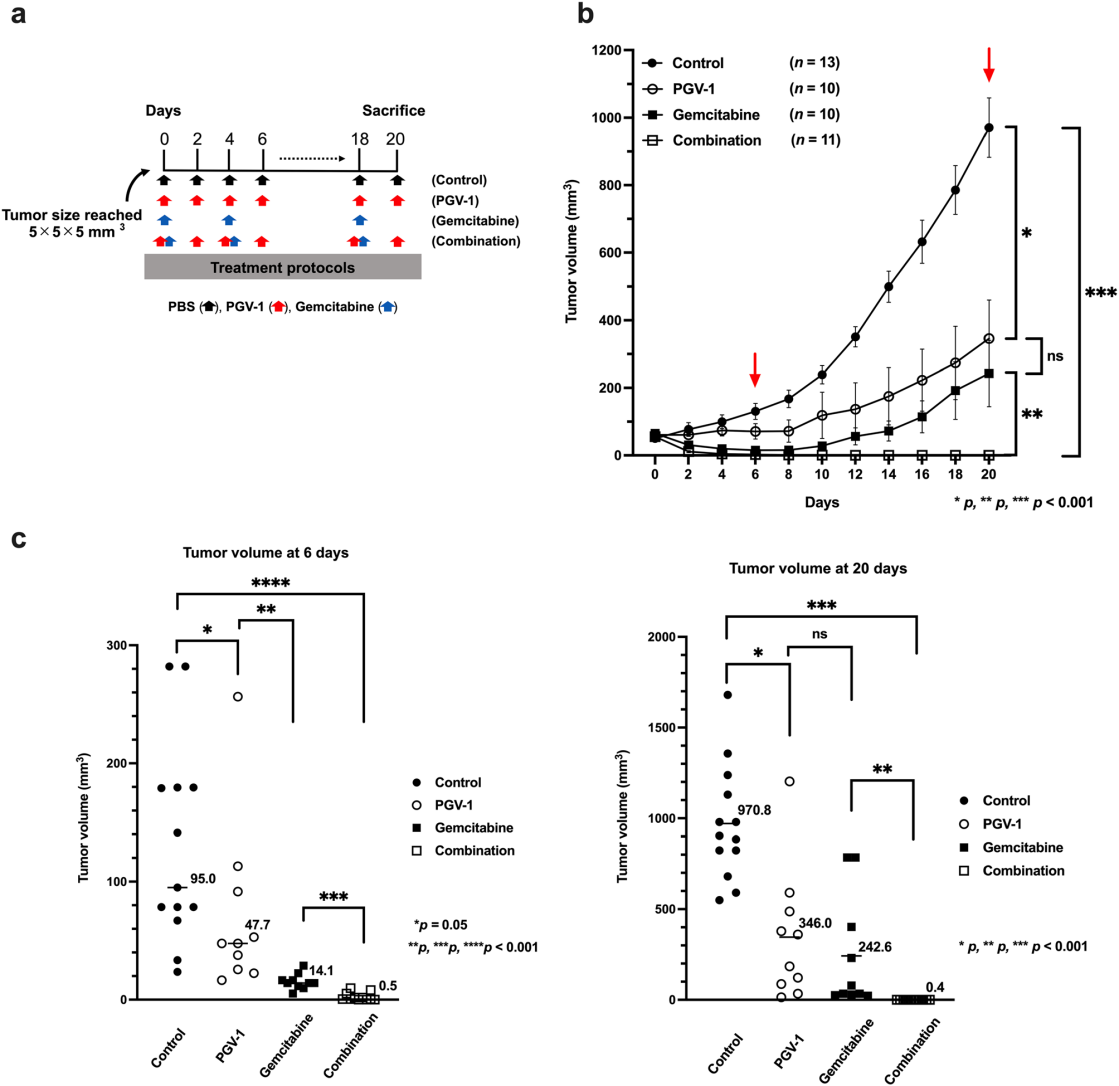
PGV-1 suppresses the growth of pre-formed tumors in CDX-M. (**a**) Experimental design to test the anti-tumor effects of PGV-1 on pre-formed tumors in CDX-M. (**b**,**c**) MIA PaCa-2 cells (1 × 10^7^ cells) were subcutaneously injected into nude mice. When tumors reached a size of 5 × 5 × 5 mm^3^, mice were randomly grouped into 4 groups and treated differently: ① control (PBS, per os), ② orally administered (p.o.) PGV-1 (25 mg/kg BW) in corn oil every 2 days, ③ intraperitoneally (i.p.) administered gemcitabine (100 mg/kg) in normal saline twice a week, and ④ the combination of PGV-1 (every two days, 25 mg/kg BW, p.o.) and gemcitabine (twice a week, 100 mg/kg BW, i.p.). Tumor volumes were measured every 2 days (**b**), and mice were sacrificed on day 20. Tumor volumes on days 6 and 20 were compared (**c**). P values were calculated with Student’s t test. Data are the average of 4 independent experiments shown as mean ± SEM (one to four mice were used for each independent experiment).

In the CDX-M experiment, PGV-1 was administered from the day of the cell inoculation; however, in clinical settings, chemotherapy is generally initiated after tumors are detected, and, thus, it is important to assess the efficacy of PGV-1 against tumors that are already formed. Since treatments with anti-cancer drugs are generally effective when pancreatic cancer is small and at the early stage, we administered PGV-1 when tumor sizes reached 5 × 5 × 5 mm^3^. In addition to PGV-1 monotherapy, we examined and compared gemcitabine monotherapy and combination therapy with both reagents under these conditions. After the inoculation, MIA PaCa-2 cells formed tumors with a size of 5 × 5 × 5 mm^3^ after five to seven days. Mice were then classified into 4 treatment groups: control, PGV-1 alone, gemcitabine alone, and the combination of PGV-1 and gemcitabine (Fig. 4a), and were treated for 20 days (Fig. 4b). The volumes of tumors in each group were compared on days 6 (Fig. 4c, left panel) and 20 (Fig. 4c, right panel), and the results obtained indicated that gemcitabine suppressed tumor growth more effectively than PGV-1 for the first 10 days (Fig. 4b,c, left panel). However, tumors reformed between the 10th and 20th days in mice treated with PGV-1 and gemcitabine alone, and, on the 20th day, the difference between PGV-1-treated and gemcitabine-treated mice became marginal (Fig. 4b,c, right panel). In contrast, combination therapy with PGV-1 and gemcitabine was excellent; the size of tumors decreased for the first 10 days and regrowth was negligible (Fig. 4b,c). Tumorigenicity in the PGV-1 group decreased to 35.6% (346.0 mm^3^ *vs.* 970.8 mm^3^; *p* < 0.001) and TGI for PGV-1 was 64.4% that in the control group after 20 days (Fig. 4b). TGI was similar in the gemcitabine group and PGV-1 group (75.0% *vs.* 64.4%; *p* = 0.500). Tumorigenicity in the combination group decreased to 0.2% that in the gemcitabine group (0.4 mm^3^ *vs.* 242.6 mm^3^; *p* < 0.001) and TGI for combination therapy was 99.8% (Fig. 4b,c). These results indicate that PGV-1 suppressed tumor regrowth as effectively as gemcitabine, and that the combination of PGV-1 and gemcitabine exerted synergistic suppressive effects on tumor growth by advanced pancreatic cancer cells (Theoretical calculation of tumor reduction rate of PGV-1 × gemcitabine was 15.7 ± 20.6%, whereas the actual reduction rate of PGV-1 together with gemcitabine was 0.0392 ± 0.0251%. The difference between these two was significant (p = 0.0103). Therefore, PGV-1 and gemcitabine act in a synergistic way).

### Establishment of the patient-derived xenograft mouse model

In contrast to experiments using cell lines, pancreatic tumors in a patient’s body have different characteristics in terms of the microenvironment and heterogeneity. Therefore, the findings of experiments using cell lines may not necessarily reflect efficacy in actual clinical cases. To examine the potential efficacy of PGV-1 using a better platform, a patient-derived xenograft mouse model was established. Since gemcitabine is a key drug for the treatment of pancreatic cancer and the ineffectiveness of gemcitabine, at least in part, is due to the acquisition of resistance, it is ideal to use specimens collected from a patient who has not yet been exposed to gemcitabine. Therefore, we selected specimens accordingly (Supplementary Table S1).

**Figure 5:**
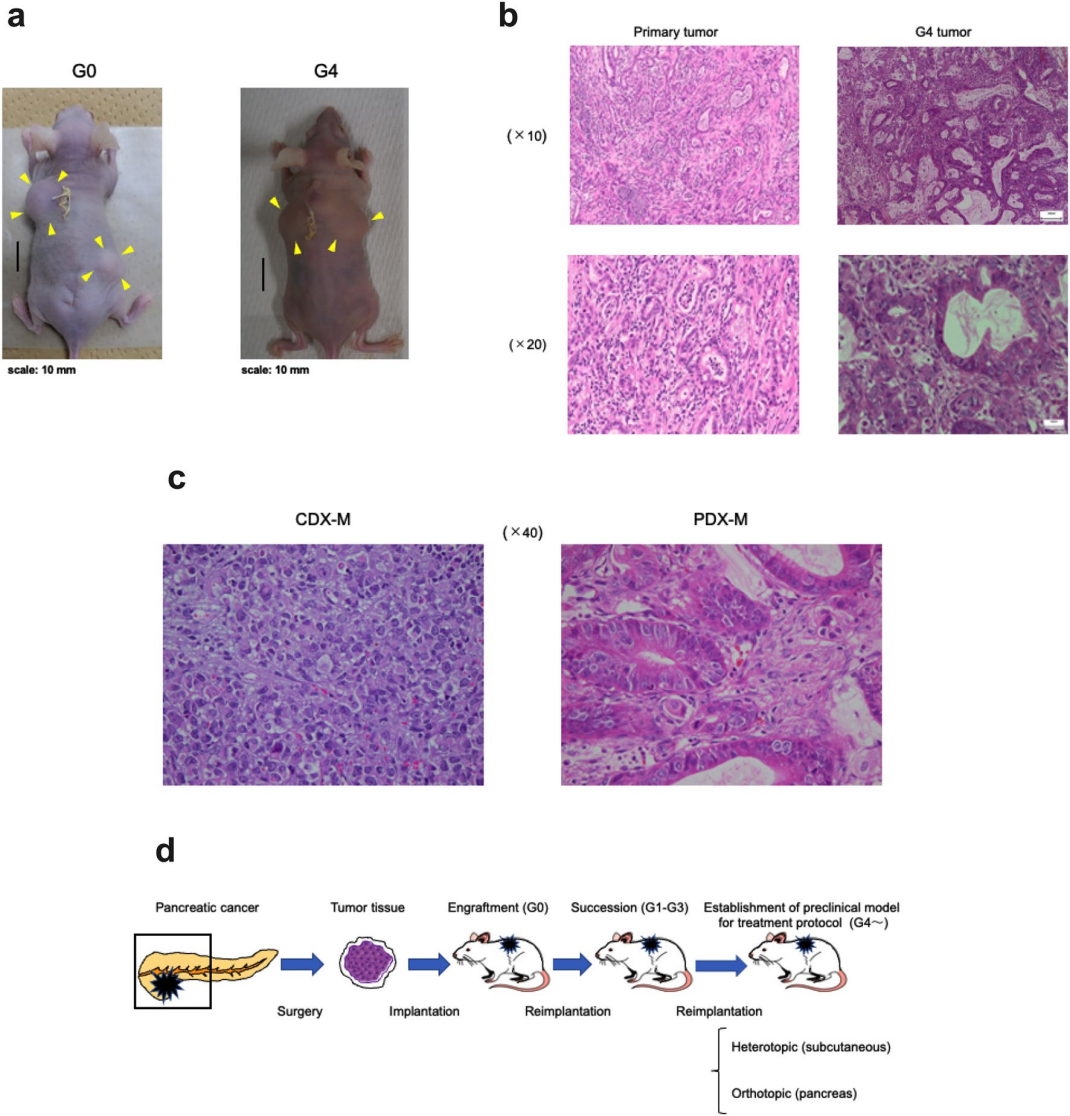
Establishment of a patient-derived xenograft mouse model. Resected specimens were cut into small pieces and directly implanted subcutaneously into the dorsal midline incisions made in nude mice with Matrigel matrix under anesthesia. (**a**) Photos of G0 and G4 in PDX-M. (**b**) Representative histological images of the primary tumor (left panels) and PDX-M of G4 (right panels) after HE staining. Low (upper panels) and high (lower panels) magnifications are shown. (**c**) Comparison of microscopic images between CDX-M and PDX-M. (**d**) Schema of the establishment of PDX-M and the experimental protocol to test anti-tumor effects.

The patient’s sample was subcutaneously embedded into nude mice and successfully passaged. The latency time for G0 tumors to reach the endpoint was 45 days, and the time of implantation to the next generation was 30–45 days after G0 (G0 and G4 mice are shown in Fig. 5a). Tumor sections of the patient’s primary tumor and PDX-M samples (G4) were stained by HE (Fig. 5b), and the histological findings of both showed moderately and poorly differentiated adenocarcinoma that were well preserved. Furthermore, in PDX-M, tumor cells were surrounded by stromal components, which was different from those in CDX-M (Fig. 5c).

Total RNA was isolated from the PDX-M sample (G4), and the genotypes of 4 major pancreas cancer driver genes (KRAS, TP53, SMAD4, and CDKN2A) were identified by RT-PCR and direct sequencing and then compared to those in the established pancreas cancer cell lines, MIA PaCa-2 and PANC-1 (Supplementary Table S2). The PDX-M sample contained point mutations in the KRAS and TP53 genes (Supplementary Fig. S3), deletions in the CDKN2A gene, and no mutations in the SMAD4 gene, which was similar to the MIA PaCa-2 and PANC-1 cell lines.

Therefore, we concluded that PDX-M samples represented the diverse clinical characteristics and well-preserved histological characteristics of the patient’s primary tumor. The flow to establish PDX-M and experiments using PDX-M are summarized in Fig. 5d.

### Anti-tumor efficacy of PGV-1 in the PDX-M assay

**Figure 6:**
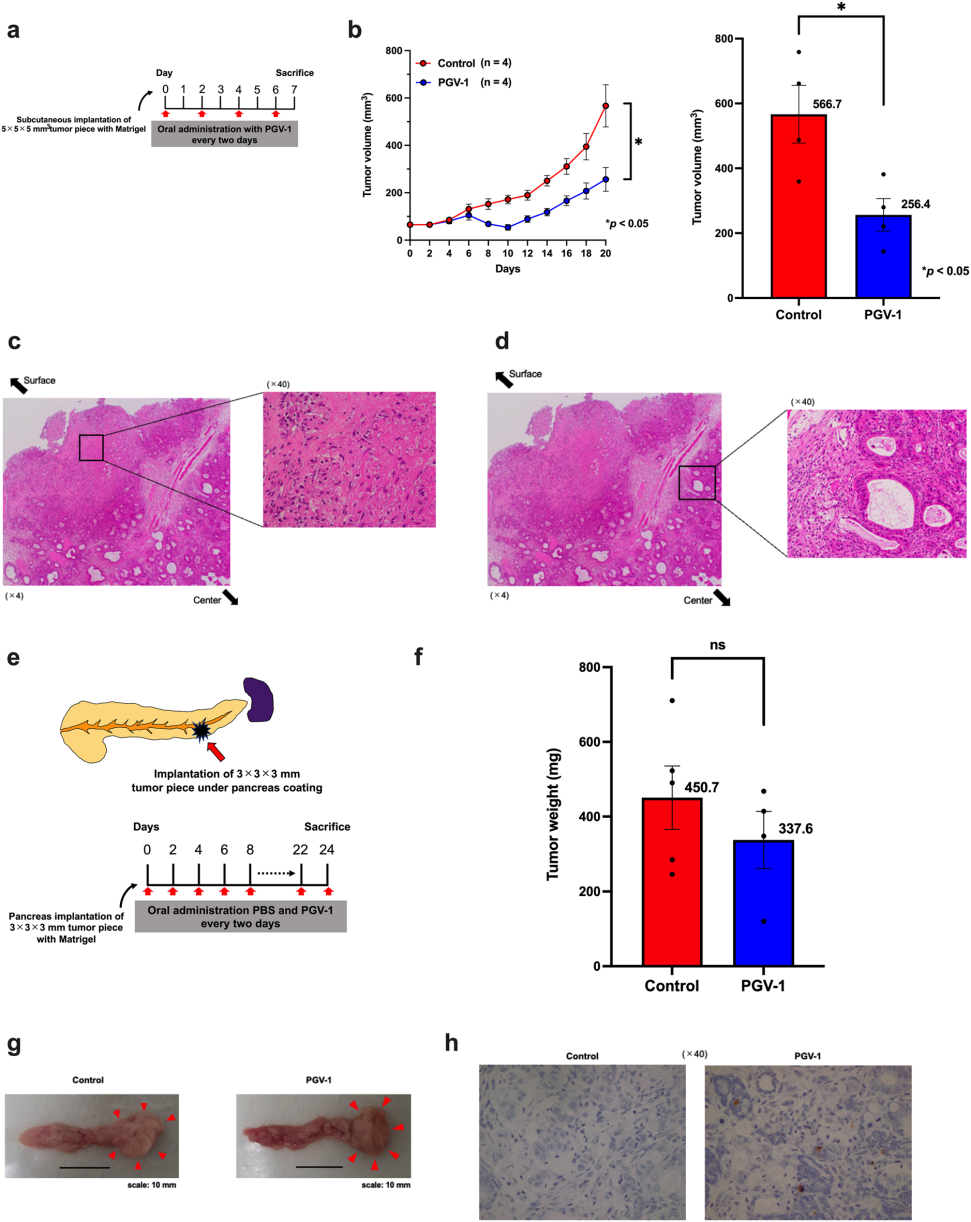
PGV-1 suppresses tumor formation at heterotopic and orthotopic sites in PDX-M. (**a**) Experimental design to test the anti-tumor effects of PGV-1 in PDX-M at the heterotopic site. (**b**–**d**) Tumor pieces (5 × 5 × 5 mm^3^) were subcutaneously implanted into nude mice, and PBS (control) and PGV-1 (25 mg/kg BW) in corn oil were orally administered every 2 days. Mice were sacrificed on the 20th day. Tumor volumes every 2 days (**b**, left panel) and on day 20 (b, right panel) are shown. Microscopic images of tumors after HE staining showed enucleation and hyalinization near the surface of the tumor (**c**), and viable cells remained in the center of tumors (**d**). (**e**) Experimental design to test the anti-tumor effects of PGV-1 in PDOX. (**f**–**h**) The abdominal cavity of mice was opened under anesthesia, and tumor pieces (3 × 3 × 3 mm^3^) were implanted into the pancreas. PBS (control) and PGV-1 (25 mg/kg BW) in corn oil were orally administered every 2 days. Mice were sacrificed on the 24th day, and the weights of tumors were measured (**f**). Photos of resected tumors are shown (**g**). Tumor samples were subjected to TUNEL staining (**h**). P values were calculated with Student’s t test. Data are the average of 4 independent experiments (**b**,**f**) shown as mean ± SEM (1–2 mice for each experiment).

A tumor piece measuring 5 × 5 × 5 mm^3^ was mixed with Matrigel and subcutaneously implanted into nude mice. PGV-1 and PBS were then orally administered from the date of implantation (Fig. 6a). After 20 days, tumors reached 566.7 ± 178.1 mm^3^ in control mice, while those in mice administered PGV-1 remained small; tumorigenicity decreased to 45.2% (256.4 ± 100.3 mm^3^ *vs.* 566.7 ± 178.1 mm^3^; *p* < 0.05) and TGI for PGV-1 was 54.8% after 20 days (Fig. 6b). These results demonstrated that PGV-1 exerted anti-tumor effects in PDX-M.

We also examined the potential efficacy of PGV-1 in histological images. Similar to experiments with CDX-M (Fig. 3), tumors were resected on the 7th day after the administration of PGV-1 and the transplantation of PDX-M samples, when the tumor growth was started to be inhibited. The microscopic examination of HE-stained tumor samples in mice treated with PGV-1 for only a limited time showed that tumor cells near the surface were affected by PGV-1, enucleated and hyalinized (Fig. 6c), whereas cells in the center remained viable (Fig. 6d), suggesting that PGV-1 acted on tumors from the surface, probably not from the inside blood vessels. These results demonstrated that PGV-1 exerted anti-tumor effects and induced apoptosis in tumor cells in PDX-M and CDX-M.

PDOX was established to reflect the behavior of pancreatic cancer cells in patients. A PDX-M sample with a size of 3 × 3 × 3 mm^3^ was implanted into the pancreas under the pancreatic coating capsule, and PGV-1 and PBS were orally administered from the date of implantation (Fig. 6e). After 24 days, mice were sacrificed, tumors were resected, and tumor volumes were compared between the control and PGV-1 groups. The mean weight of tumors was lower in the PGV-1 group than in the control group (450.7 ± 189.8 mg *vs.* 337.6 ± 153.0 mg; *p* = 0.367), but was not significantly different, which is in contrast to the results obtained from CDX-M (Fig. 6f,g). TUNEL staining of samples showed that PGV-1 induced apoptosis in tumor cells more effectively than in the control group (Fig. 6h). Therefore, although PDX-M at the heterotopic site showed the histological anti-tumor effects of PGV-1 on tumor cells similar to that in CDX-M, no significant difference was observed between the control and PGV-1 groups in PDOX.

### Effects of PGV-1 on pre-formed tumors in the PDX-M assay

**Figure 7:**
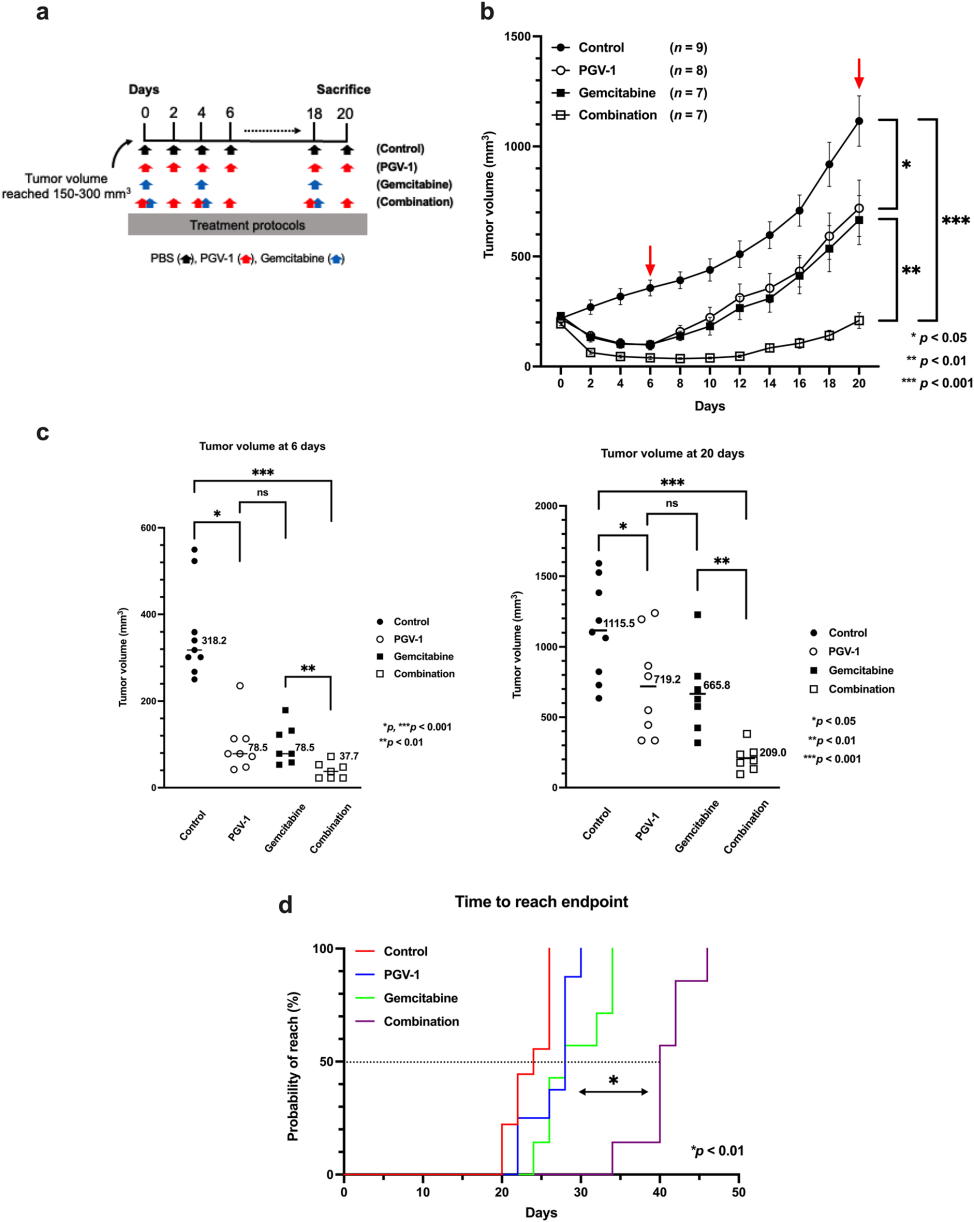
PGV-1 suppresses the growth of pre-formed tumors in PDX-M. (**a**) Experimental design to test the anti-tumor effects of PGV-1 on pre-formed tumors in PDX-M. (**b**–**d**) Tumor pieces were subcutaneously implanted into nude mice. When tumors reached a size of 150–300 mm^3^, mice were randomly grouped into 4 groups and treated differently: ① control (PBS, per os), ② PGV-1 (25 mg/kg BW p.o.) in corn oil every 2 days, ③ gemcitabine (100 mg/kg i.p.) in normal saline twice a week, and ④ the combination of PGV-1 (every two days, 25 mg/kg BW, p.o.) and gemcitabine (twice a week, 100 mg/kg BW, i.p.). Tumor volumes were measured every 2 days (**b**), and mice were sacrificed on day 20. Tumor volumes on days 6 and 20 were compared (**c**). The number of days until tumor volumes reached 1500 mm^3^ (the endpoint) is shown (**d**). P values were calculated with Student’s t test. Data are the average of 4 independent experiments shown as mean ± SEM (1–2 mice were used for each independent experiment).

PGV-1 was administered when tumor volumes reached 150–300 mm^3^ to investigate its efficacy against pre-formed tumors. In PDX-M, tumor volumes reached 150–300 mm^3^ seven to ten days after the date of implantation. Mice were then classified into the following treatment groups: control, PGV-1 alone, gemcitabine alone, and the combination of PGV-1 and gemcitabine (Fig. 7a). After 20 days of treatment, the mean volume of tumors reached 1115.5 mm^3^ in control group, and mice were sacrificed (Fig. 7b). Tumor volumes on the 6th and 20th day in each group were compared in Fig. 7c (left panel; at 6th day, right panel; on the 20th day). Tumorigenicity in the PGV-1 group decreased to 64.5% that in the control group (719.2 mm^3^ *vs.* 1115.5 mm^3^; *p* < 0.05), and TGI for PGV-1 was 35.5% on the 20th day (Fig. 7c). TGI in the gemcitabine group was similar to that in the PGV-1 group (40.3% *vs.*35.5%; *p* = 0.762) (Fig. 7c). These results demonstrated that PGV-1 alone exerted similar anti-tumor effects on pre-formed tumors in PDX-M as gemcitabine alone. Furthermore, tumorigenicity in the combination group decreased to 31.4% that of gemcitabine alone (209.0 mm^3^ *vs.* 665.8 mm^3^; *p* < 0.01) and TGI for combination therapy was 81.3% (Fig. 7c), indicating that the combination of PGV-1 and gemcitabine exerted strong synergistic effects to suppress tumor growth in PDX-M (Theoretical calculation of tumor reduction rate of PGV-1 + gemcitabine was 47.9 ± 33.8%, whereas the actual reduction rate of PGV-1 together with gemcitabine was 16.1 ± 7.93%. The difference between these two was significant (p = 0.0208). Therefore, PGV-1 and gemcitabine act in a synergistic way).

We also investigated the extent to which each treatment protocol prolonged survival. The endpoint was set as the time when the tumor volume reached 1500 mm^3^, and the number of days was counted in each group. In mice treated with PGV-1 alone, the median survival time (MST) was 17% longer than that of control mice (28 days *vs.* 24 days; *p* < 0.05) (Fig. 7d). No significant differences were observed in MST between mice treated with PGV-1 alone and gemcitabine alone (28 days *vs.* 28 days; *p* = 0.183). However, the MST of mice treated with the combination of PGV-1 and gemcitabine was 43% longer than that of mice treated with gemcitabine alone (40 days *vs.* 28 days; *p* < 0.001), and was 67% longer than that of control mice (40 days *vs.* 24 days; *p* < 0.001). These results indicate that PGV-1 is a good candidate anti-cancer drug for pancreatic cancer, and based on the strong synergistic effects of the combination of PGV-1 and gemcitabine, combination therapy with PGV-1 and gemcitabine may effectively prolong survival times in clinical cases of advanced pancreatic cancer.

### Side effects of PGV-1

**Figure 8:**
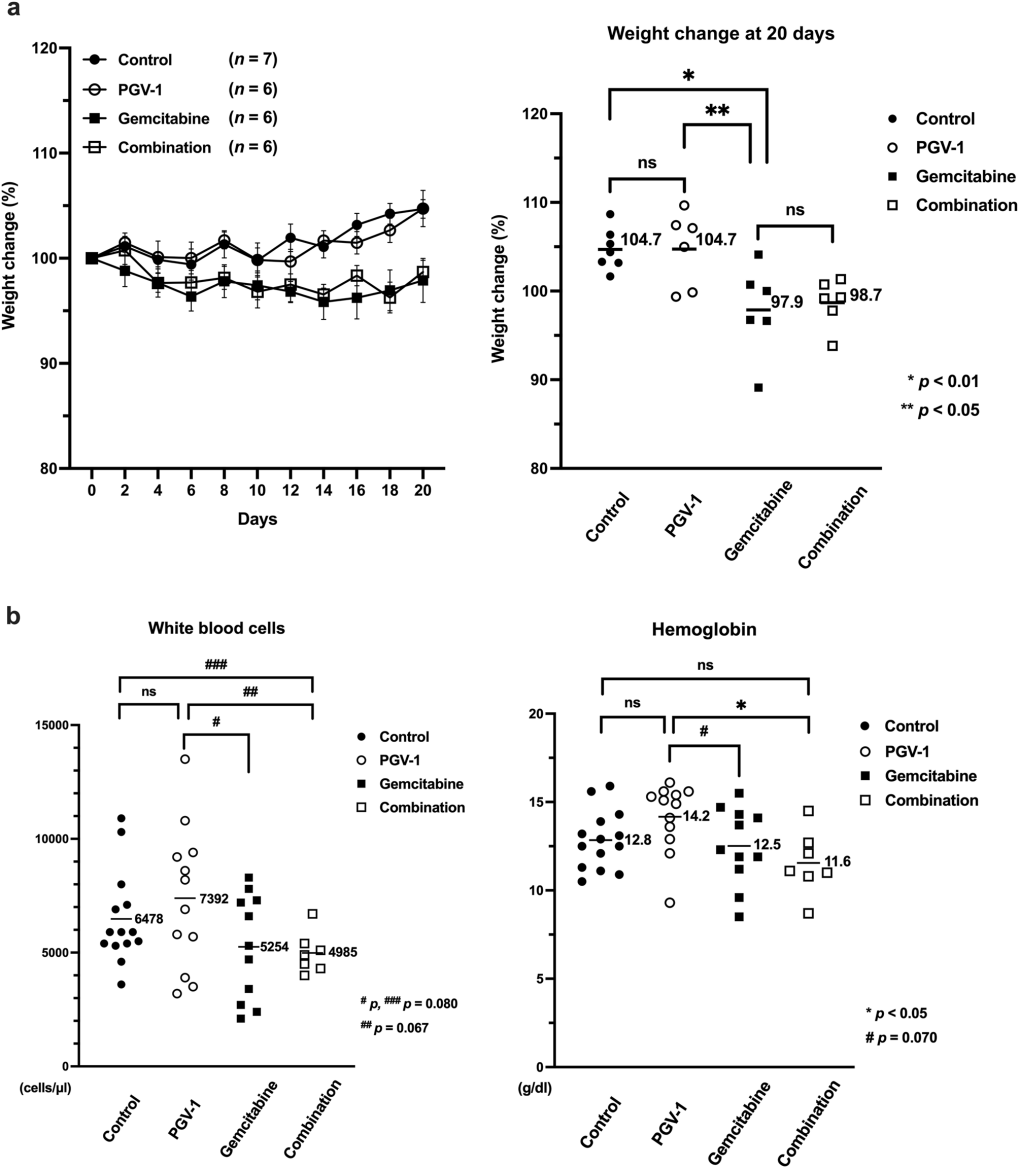
Examination of side effects by PGV-1. In mice treated as described in Fig. 7, body weights were measured every 2 days (**a**, left panel), and those at day 20 (**a**, right panel) are shown. White blood cell counts (**b**, left panel) and hemoglobin concentrations (**b**, right panel) in peripheral blood were measured two weeks after administration. P values were calculated with Student’s t test. Data are the average of 4 independent experiments shown as mean ± SEM (1–2 mice were used for each independent experiment). White blood cell counts and hemoglobin concentrations were measured twice per one mouse.

We investigated side effects, such as myelosuppression and weight loss, in the xenograft mouse model implanted with pancreatic cancer cells and in mice treated with the combination of PGV-1 and gemcitabine. The body weights of mice in each group were measured every two days. On the 20th day, no significant differences were observed between the control and PGV-1 groups (104.7% *vs.* 104.7%) or between the gemcitabine and combination groups (97.9.% *vs.* 98.7%) (Fig. 8a,b). A significant difference was noted between the control and gemcitabine groups (104.7% *vs.* 97.9%; *p* < 0.01) and between the PGV-1 and gemcitabine groups (104.7% *vs.* 97.9%; *p* < 0.05) (Fig. 8a). These results showed that weight loss was a side effect of gemcitabine, but not PGV-1.

Side effects on bone marrow were also examined; white blood cell counts and hemoglobin concentrations in peripheral blood were measured two weeks after the administration of treatments (Fig. 8b). No significant differences were observed in white blood cell counts (6478 cells/μl *vs.* 7392 cells/μl) or hemoglobin concentrations (12.8 g/dl *vs.* 14.2 g/dl) between the control and PGV-1 groups, while white blood cell counts and hemoglobin concentrations were lower in the combination group than in the PGV-1 group (white blood cells: 7392 cells/μl *vs.* 4985 cells/μl; *p* = 0.067, and hemoglobin concentrations: 14.2 g/dl *vs.* 11.6 g/dl; *p* < 0.05, respectively), which may have been due to the side effects of gemcitabine. These side effects of gemcitabine were not accelerated in the presence of PGV-1. In summary, PGV-1 did not reduce body weight or white blood cell counts and hemoglobin concentrations in peripheral blood. Any other differences in behavior and macroscopic appearance were absent in PGV-1-treated mice. Furthermore, no synergistic enhancement in side effects was observed with combination therapy.

## Discussion

Pancreatic cancer is more malignant and aggressive than other cancers^25^. In cases of locally advanced or unresectable pancreatic cancer, chemotherapy is the mainstay of treatment. Surgery following neoadjuvant chemoradiotherapy has recently been used as the standard treatment, even for resectable pancreatic cancer^26^. Therefore, chemotherapy is expected to play a more central role in the treatment of pancreatic cancer in the future. In clinical cases, pancreatic cancer cells are histologically surrounded by a dense stroma and are categorized as hypovascular tumors. These characteristics have been proposed as the cause of the poor prognosis of patients with pancreatic cancer. The results of the PDX-M assay revealed that apoptosis was induced in cells on the surface of tumors treated with PGV-1. On the other hand, viable tumor cells remained in the center of tumors similar to tumors in untreated mice. Furthermore, the results of the PDOX assay revealed no significant difference in terms of tumor weights between the control and PGV-1 groups. These results indicated that part of the tumor or the implantation site was affected by the low perfusion of blood. Moreover, histological findings obtained from the HE staining of tumors in the PDX-M assay revealed not only cancer cells, but also stroma components. Therefore, for a chemotherapeutic drug to reach and penetrate pancreatic cancer cells, a sufficient amount of the drug needs to be administered. However, clinical trials demonstrated that higher doses of chemotherapeutic drugs were associated with an increased incidence of side effects with negligible therapeutic benefits^27,28^. Therefore, transformative chemotherapeutic agents with sufficient efficacy and fewer side effects need to be established in order to improve the prognosis of patients with pancreatic cancer.

Pancreatic cancer cells in a patient’s body differ from pancreas cancer cell lines cultured in vitro in terms of heterogeneity and the microenvironment, and, thus, it is crucial to assess the efficacy of novel compounds with discretion. The results of the CDX-M assay showed that PGV-1 suppressed tumor growth and TGI was 96.6%. In contrast, PGV-1 inhibited tumor growth and TGI decreased to 54.8% in the PDX-M assay. In the CDX-M assay with pre-formed tumors, TGI for PGV-1 monotherapy, gemcitabine monotherapy, and combination therapy with PGV-1 and gemcitabine were 64.4, 75.0, and 99.8%, respectively, whereas in the PDX-M assay with pre-formed tumors, TGI were 35.5, 40.3, and 81.3%, respectively. These results clearly showed differences in the efficacy of drugs between the CDX-M and PDX-M assays, and indicate that the results obtained from cell line-based experiments are not sufficient to evaluate the efficacy of chemotherapeutic candidates.

A previous study reported that neoadjuvant chemotherapy induced somatic mutations^29^. Therefore, it is difficult to use chemotherapy-treated samples for the accurate evaluation of chemotherapeutic candidates because the additional mutations caused by chemotherapy may alter drug sensitivity. Regarding gemcitabine, since gemcitabine treatments induce resistance, it is inappropriate to use gemcitabine-treated specimens to evaluate the efficacy of gemcitabine. Resected samples of pancreatic cancer without neoadjuvant chemotherapy were used in the present study to establish PDX-M, thereby avoiding these issues. Moreover, the success rate of engraftment for PDX-M was shown to decrease to approximately 30% when a resected sample of pancreatic cancer that had been treated with neoadjuvant chemotherapy was used^30^. Therefore, neoadjuvant chemotherapy may induce apoptosis in tumor cells and fibrosis. In terms of accurate evaluations and successful establishment, it is ideal to use a resected sample that has never been exposed to neoadjuvant chemotherapy.

In the CDX-M assay with pre-formed tumors, tumors were significantly larger in PGV-1-treated mice than in gemcitabine-treated mice after six days, whereas no significant differences were observed in tumor volumes between mice treated with PGV-1 and gemcitabine in the PDX-M assay with pre-formed tumors on day 6. These results may be attributed to different genetic mutations in the CDX- M and PDX-M assays; however, the genotypes of major pancreatic cancer driver genes did not markedly differ between cells used in the CDX- M and PDX-M assays (Supplementary Table 2), suggesting that differences other than genetic mutations may have contributed to these responses.

In translational cancer research, many chemotherapeutic candidates have been investigated for use in combination therapy with gemcitabine, and some have successful exerted synergistic effects as in preclinical models^31–33^. Recent studies demonstrated that combination therapy with curcumin/curcumin-related compounds and gemcitabine exerted stronger anti-tumor effects than monotherapy with each reagent^34,35^, while other studies reported no synergistic effects for combination therapy with gemcitabine^36^. The present results from CDX-M and PDX-M assays showed that combination therapy with PGV-1 and gemcitabine suppressed tumor growth more effectively than each monotherapy.

There is currently no explanation for the high efficacy of combination therapy with PGV-1 and gemcitabine; however, PGV-1 may inhibit the acquisition of gemcitabine resistance in addition to its tumor-suppressing ability. PGV-1 and gemcitabine may also act on different points of the cell cycle (PGV-1 for the M phase and gemcitabine for the S phase), thereby preventing resistance. The combination of reagents that inhibit different points of the cell cycle, namely, the combination of gemcitabine and nab-paclitaxel, has been applied. However, since nab-paclitaxel causes severe neutropenia and peripheral neuropathy by inhibiting the effects of microtubulin, severe side effects caused by both drugs are intolerable. PGV-1 did not induce side effects, such as weight loss and myelosuppression. Furthermore, it did not enhance side effects caused by gemcitabine. Therefore, PGV-1 is a good candidate for combination therapy with gemcitabine. Furthermore, PGV-1 was effective when orally administered, which reduces the burden on patients. In clinical cases, PGV-1 as monotherapy is suitable for the prevention of postoperative recurrence in patients with pancreatic cancer. PGV-1 in combination therapy with gemcitabine is suitable for locally advanced or unresectable cases of pancreatic cancer. In conclusion, we conducted experiments using multiple xenograft assays to investigate the potential efficacy of PGV-1 as both monotherapy and combination therapy. PGV-1 is a highly potent chemotherapeutic candidate for pancreatic cancer. We propose that PGV-1 needs to be pharmaceutically developed as an orally administered drug for the treatment of pancreatic cancer.

## Materials and methods

### Compounds

PGV-1 ((2E,5E)-2-[(4-hydroxy-3,5-dimethylphenyl) methyldene]-5-[(3-methoxy-4,5-dimethylphenyl) methylidene] cyclopentan-1-one) (purity ≥ 99.8%) was obtained from the Curcumin Research Center, Faculty of Pharmacy, Universitas Gadjah Mada. Gemcitabine was purchased from Nippon Kayaku (Tokyo, Japan). Matrigel matrix was purchased from Corning Company (NY, USA).

### Cell culture

Human cancer cell lines (MIA PaCa-2 and PANC-1) were cultured in Dulbecco’s modified Eagle’s medium (DMEM) supplemented with fetal bovine serum and antibiotics as previously described^11,13^. Viable cells were counted by the trypan blue exclusion method.

### Animal experiments

Mice were kept under specific pathogen-free conditions (the Animal Experimentation Facility of NAIST), and all methods were performed in accordance with NAIST guidelines and regulations. The experimental protocols used in the present study were approved by the NAIST Institutional and Licensing Committees. The study is reported in accordance with ARRIVE guidelines. BALB/c nude mice (6–8 weeks old) were used in experiments. Cells (1 × 10^7^ cells) were subcutaneously or orthotopically injected into nude mice, and PGV-1 (25 mg/kg BW) or PBS was orally administered. Gemcitabine (100 mg/kg) was intraperitoneally administered. The longest and shortest tumor diameters were measured at the indicated times, and tumor volumes were calculated as follows: V = 3.14 × LD × (SD)^2^/6, where V represents the tumor volume, and LD and SD are the longest and shortest tumor diameters, respectively^13,37^. When necessary, after tumor volumes reached 5 × 5 × 5 mm^3^, mice were randomly grouped into 4 groups and treated with ① PBS (control), ② PGV-1 alone, ③ gemcitabine alone, and ④ the combination of PGV-1 and gemcitabine. After mice were sacrificed, tumors were resected, and the tumor weights were measured. The tumor growth inhibition index (TGI) was calculated as follows: TGI = (Average tumor volume or weight in the control group − Average tumor volume or weight in the treatment group)/Average tumor volume or weight in the control group × 100%. For evaluation of synergy, the tumor reduction rate caused by PGV-1 alone in each individual mouse was multiplied by that of gemcitabine alone in each individual mouse, and the average of double tumor reduction rate was calculated (theoretical calculation). If the actual reduction rate of PGV-1 together with gemcitabine is significantly smaller than the theoretical calculation of double tumor reduction rate (a *p* value of < 0.05), we conclude that the effect is synergistic.

### Histological assessment

Primary tumor tissues and PDX-M samples were fixed in 10% formalin, embedded in paraffin, and then sectioned for hematoxylin and eosin (HE) staining. TUNEL staining was performed to assess apoptosis in tumor sections. Cells within five randomly selected fields of each sample were analyzed at a high magnification and the apoptosis index was calculated as the number of TUNEL-positive cells per two hundred cells. In addition, cells with fragmented nuclei were not positive for TUNEL, but were judged to be undergoing apoptosis and were counted as apoptosis-positive cells.

### Measurement of DNA contents

Tumors detected at the subcutaneous site were resected, cut into small pieces in DMEM, and filtered, and cells were then fixed on glass slides by the cytospin technique. After fixing with 4% paraformaldehyde, chromosomal DNA was stained with Hoechst 33342 and observed under a confocal microscopy. DNA contents in both groups were measured using ImageJ software.

### Surgical specimens of pancreatic cancer

Patient characteristics are summarized in Supplementary Table S1. Contrast-enhanced computed tomography of the patient revealed a 50-mm pancreatic head tumor with poor contrast (see Supplementary Fig. S1). The tumor was histologically judged to be adenocarcinoma via endoscopic ultrasound-guided fine needle aspiration, and the patient was diagnosed with pancreatic cancer. Pancreaticoduodenectomy without neoadjuvant chemoradiotherapy was performed for radical resection at Nara Medical University. The resected specimen is shown in Supplementary Fig. S2. These experiments were approved by the Local Ethics Committee on Clinical Investigations of Nara Medical University (No. 2943), and written informed consent was obtained from the patient.

### Establishment of PDX-M and evaluation for treatment

Tumor tissue was rinsed with PBS three times, preserved in DMEM immediately after resection, and transferred to Nara Institute of Science and Technology within 2 h. Resected specimens were cut into small pieces (1–3 mm^3^) and directly implanted into nude mice. Mice were anesthetized using isoflurane (2.2–2.5%) and small dorsal midline incisions (8–10 mm) were made. Tumor tissues with 100 μl of Matrigel matrix were placed in lateral subcutaneous pockets and the incisions were closed. At this point, mice were defined as G0. When tumor volumes reached 1500 mm^3^, mice were sacrificed and tumor tissues were harvested and re-implanted into new mice for the next passage using the same protocol. PDX-M samples, measuring 5 × 5 × 5 mm^3^ and 3 × 3 × 3 mm^3^, were implanted into the subcutaneous space and pancreas from G4, respectively, to investigate the efficacy of each drug.

### Identification of genotypes of pancreatic cancer cells

Tumor tissues were cut into small pieces, and total RNA was isolated using the ISOGEN reagent (Nippon Gene) and then reverse transcribed using RNase-free Superscript reverse transcriptase (Invitrogen) according to the manufacturer’s instructions. The coding sequences of human K-RAS, TP53, SMAD4, and CDKN2A were amplified by PCR using total cDNA as the template. PCR fragments were directly sequenced using the same primers as those used for PCR. The following oligonucleotide primers specific to human K-RAS, TP53, SMAD4, and CDKN2A were used: human K-RAS, 5’-CCT GCT GAA AAT GAC TGA ATA-3' (sense) and 5’-CAT CAT CAA CAC CCA GAT TAC-3’ (antisense); human TP53, 5’-GCA TTC TGG GAC AGC CAA GT-3' (sense) and 5’-TCA GCT CTC GGA ACA TCT CG-3’ (antisense); human SMAD4#1, 5’-CCT GTT CAC AAT GAG CTT GC-3' (sense) and 5’-CAC CAT CCT GAT AAG GTT AAG G -3’ (antisense); human SMAD4#2, 5’-GCC CAG GTT ATC CTG AAT AC-3' (sense) and 5’-CCA TCC AAT GTT CTC TGT ATG-3’ (antisense); human CDKN2A, 5’-CAT GGA GCC TTC GGC TGA CT-3' (sense) and 5’-CCG AGG TTT CTC AGA GCC TCT C-3’ (antisense).

### Each in vivo treatment protocol

PGV-1 dissolved in corn oil was orally administered at a concentration of 25 mg/kg every two days. Gemcitabine was intraperitoneally administered at a concentration of 100 mg/kg twice a week. Combination therapy was defined as the combination of these two drugs. When subcutaneous tumor volumes reached 5 × 5 × 5 mm^3^ in CDX-M or 150–300 mm^3^ in PDX-M, the administration of each drug was initiated. Tumor volumes were measured every two days.

### Statistical analysis

Continuous variables were compared using the Student’s *t*–test or Mann–Whitney *U* test. All statistical analyses were performed using EZR version 1.29 (Saitama Medical Centre, Jichi Medical University, Saitama, Japan). A *p* value of < 0.05 denoted significance and values were added to every figure. Graphs were made using Graph Pad Prism version 9 for Mac OS (Graph Pad Software, San Diego, California, USA).

## Supplementary Information

Supplementary Information.

## Data Availability

All data generated or analyzed during this study are included in this published article and its supplementary information files.
