# Supplementary material for: Preclinical evaluation of pentagamavunone-1 as monotherapy and combination therapy for pancreatic cancer in multiple xenograft models
Source: Sci Rep. 2022 Dec 27;12:22419. doi: 10.1038/s41598-022-26863-y (PMC9794715; doi:10.1038/s41598-022-26863-y)
Supplement: Supplementary file 1 — Supplementary Information. [file 41598_2022_26863_MOESM1_ESM.pdf]

Preclinical evaluation of pentagamavunone-1 as monotherapy and combination therapy for pancreatic cancer in multiple xenograft models

Naoki Kamitani[1], Ikuko Nakamae[2], Noriko Yoneda-Kato[2], Jun-ya Kato[2]*, and Masayuki Sho[1]

Affiliations and addresses:

1) Department of Surgery, Nara Medical University, 840 Shijo-Cho, Kashihara, Nara 634-8522, Japan

2) Laboratory of Tumor Cell Biology, Division of Biological Science, Graduate School of Science and Technology, Nara Institute of Science and Technology, 8916-5, Takayama, Ikoma, Nara 630-0101, Japan

***Address correspondence to:**

Jun-ya Kato,

Laboratory of Tumor Cell Biology, Division of Biological Science,

Graduate School of Science and Technology, Nara Institute of Science and Technology,

8916-5, Takayama, Ikoma, Nara 630-0101, Japan.

Phone: +81-743-72-5510, E-mail: jkata@bs.naist.jp

Supplementary Figure Legends

Supplementary Figure S1

Contrast-enhanced computed tomography of the patient. A tumor with poor contrast was detected in the pancreas head (yellow arrowhead).

Supplementary Figure S2

Resected specimen of pancreatic cancer.

Supplementary Figure S3

Sequences of mutated sites in KRAS and TP53 genes in the PDX-M sample.

Supplementary Figures

Supplementary Figure S1

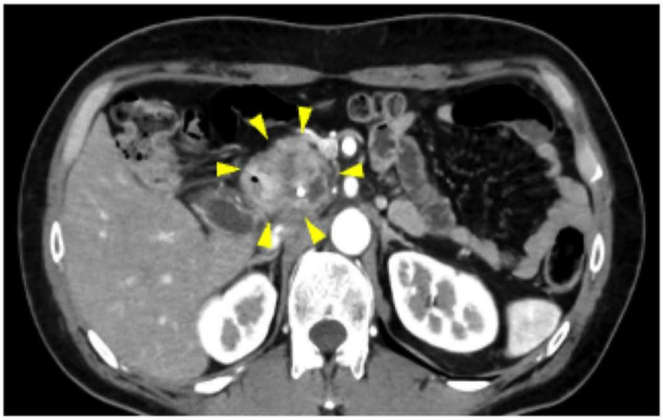

Supplementary Figure S2

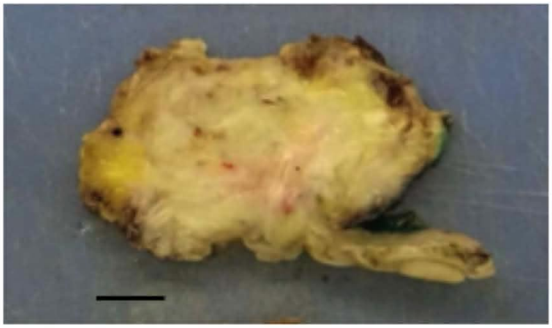

scale: 10 mm

Supplementary Figure S3

Seq Result

KRas

c.35G>A , p.G12D

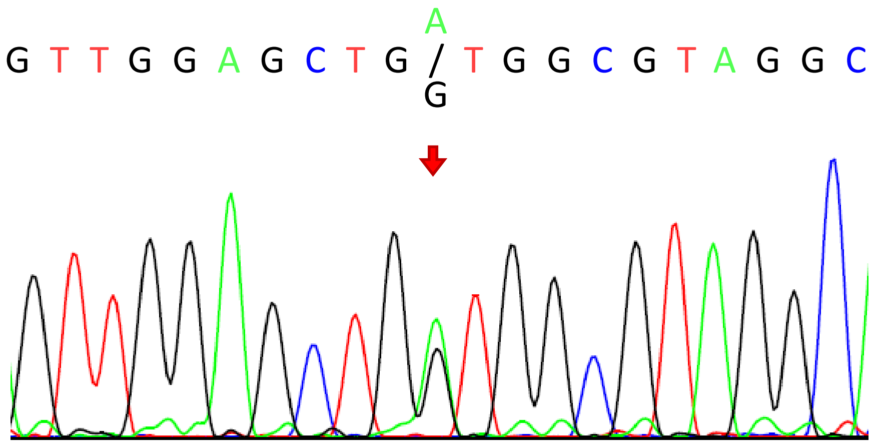

TP53

c.743G>A , p.R248Q

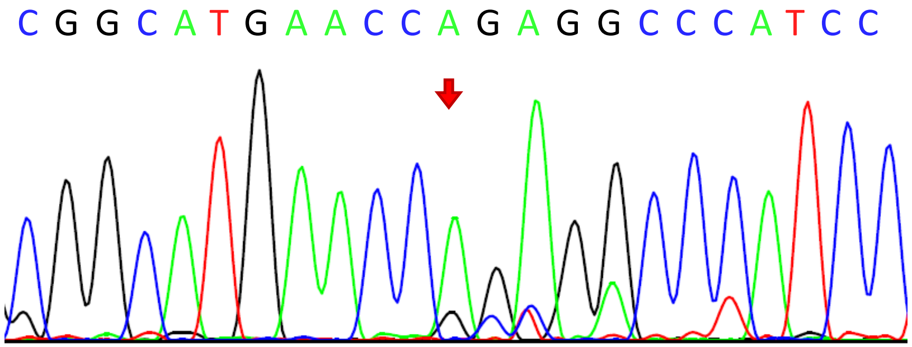

Supplementary Table S1 Characteristics of the patient for PDX model of pancreatic cancer

	Present case
Age	48
Gender	Male
Tumor location	Head
Preoperative serum CEA (ng/mL)	5.7
Preoperative serum CA19-9 (U/mL)	415
Preoperative serum DUPAN-2 (U/mL)	1590
Neoadjuvant therapy	absent
Tumor size (mm)	55
TNM staging (UICC 8th edition)	T3N1bM0, cStage IIb
Tumor differentiation	moderately and poor differentiated type
Pathological lymphnode metastasis	positive
Pathological lymphvascular invasion	positive

Abbreviations: *CEA*, Carcinomaembryonic antigen; *CA19-9*, Carbohydrate antigen 19-9; *DUPAN-2*, Duke pancreatic cancer associated antigen-2; *UICC*, Union for international cancer control

Supplementary Table S2 Somatic mutations of patient's sample, PDX-M and pancreatic cancer cell-lines.

	PDX-M	MIA PaCa-2	PANC-1
KRAS	Missense (G12D)	Missense (G12C)	Missense (G12D)
TP53	Missense (R248Q)	Missense (R248W)	Missense (P72R, R273H)
SMAD4	No mutation	No mutation	No mutation
CDKN2A	Deletion	Deletion	Deletion

Abbreviations: *PDX-M*, Patients' derived xenograft mouse mode
